# Squalene synthase promotes the invasion of lung cancer cells via the osteopontin/ERK pathway

**DOI:** 10.1038/s41389-020-00262-2

**Published:** 2020-08-29

**Authors:** Yi-Fang Yang, Yu-Chan Chang, Yi-Hua Jan, Chih-Jen Yang, Ming-Shyan Huang, Michael Hsiao

**Affiliations:** 1 Department of Medical Education and Research, Kaohsiung Veterans General Hospital, Kaohsiung, Taiwan; 2 Department of Biomedical Imaging and Radiological Sciences, National Yang-Ming University, Taipei, Taiwan; 3 Genomics Research Center, Academia Sinica, Taipei, Taiwan; 4 Division of Pulmonary and Critical Care Medicine, Department of Internal Medicine, Kaohsiung Medical University Hospital, Kaohsiung Medical University, Kaohsiung, Taiwan; 5 Department of Internal Medicine, Kaohsiung Municipal Ta-Tung Hospital, Kaohsiung Medical University, Kaohsiung, Taiwan; 6 Department of Respiratory Therapy, College of Medicine, Kaohsiung Medical University, Kaohsiung, Taiwan; 7 Department of Internal Medicine E-DA Cancer Hospital School of Medicine, I-Shou University Kaohsiung, Kaohsiung, Taiwan; 8 Department of Biochemistry, College of Medicine, Kaohsiung Medical University, Kaohsiung, Taiwan

**Keywords:** Oncogenes, Lipid signalling

## Abstract

Cholesterol is the major component of lipid rafts. Squalene synthase (SQS) is a cholesterol biosynthase that functions in cholesterol biosynthesis, modulates the formation of lipids rafts and promotes lung cancer metastasis. In this study, we investigated the lipid raft-associated pathway of SQS in lung cancer. Gene expression microarray data revealed the upregulation of secreted phosphoprotein 1 (SPP1; also known as osteopontin, OPN) in CL1-0/SQS-overexpressing cells. Knockdown of OPN in SQS-overexpressing cells inhibits their migration and invasion, whereas an OPN treatment rescues the migration and invasion of SQS knockdown cells. High OPN expression is associated with lymph node status, advanced stage and poor prognosis in patients with lung cancer. Moreover, patients with high SQS expression and high OPN expression show poor survival compared with patients with low SQS expression and low OPN expression. SQS induces the phosphorylation of Src and ERK1/2 via OPN, resulting in increased expression of MMP1 and subsequent metastasis of lung cancer cells. Based on our findings, SQS expression increases the expression of OPN and phosphorylation of Src through cholesterol synthesis to modulate the formation of lipid rafts. SQS may represent a therapeutic strategy for lung cancer.

## Introduction

Lung cancer is one of the most common malignancies both in Taiwan and in many other countries. It remains the leading cause of cancer-related death, with more than 1.3 million people dying of the disease annually^1^. Lung cancer is classified according to the histological type into small cell carcinoma and non-small cell lung carcinoma (NSCLC); NSCLC accounts for approximately 80% of all lung cancer cases^2,3^. Unfortunately, approximately 70% of all patients who are newly diagnosed with NSCLC present with locally advanced or metastatic disease and require systemic treatment. Patients with NSCLC often have a poor prognosis, and the 5-year survival rate of patients with all stages of the disease combined is only 15%^3–5^. The high mortality rate of NSCLC is attributed to its high rate of metastasis^6^. According to previous studies, enzymes involved in the cholesterol biosynthesis pathway are upregulated in lung cancer, and strategies targeting cholesterol biosynthesis enzymes inhibit invasion and migration, indicating that enzymes involved in cholesterol biosynthesis play an important role in cancer progression^7^. Moreover, cholesterol also modulates the formation of lipid rafts (rafts) in the cell membrane. The functions of rafts include the assembly of signaling molecules, modulation of membrane fluidity and membrane protein or receptor exchange^8–10^. Rafts also provide platforms for the coordination of different molecules^11,12^. The cholesterol biosynthesis enzyme squalene synthase (SQS) modulates raft composition and promotes metastasis in lung cancer by inducing clustering of rafts in the cell membrane, leading to an enrichment of the TNF-α receptor in rafts^7^.

Secreted phosphoprotein 1 (SPP1, also known as osteopontin, OPN) was identified and subjected to further analysis due to its ability to promote the invasion and metastasis of various types of cancer^13^. OPN was first described as a glycophosphoprotein that is secreted from malignant epithelial cells^14^. The roles of OPN in cancer progression include cell adhesion, chemotaxis, invasion, migration, and anchorage-independent growth of tumor cells^15^. In our previous study, the highest expression of SPP1 was observed in SQS-overexpressing cells^7^. However, the mechanism by which SQS modulates OPN expression to regulate the malignant phenotype has not been clearly elucidated.

## Results

### SQS expression is correlated with poor survival rates in lung cancer patients

**Fig. 1:**
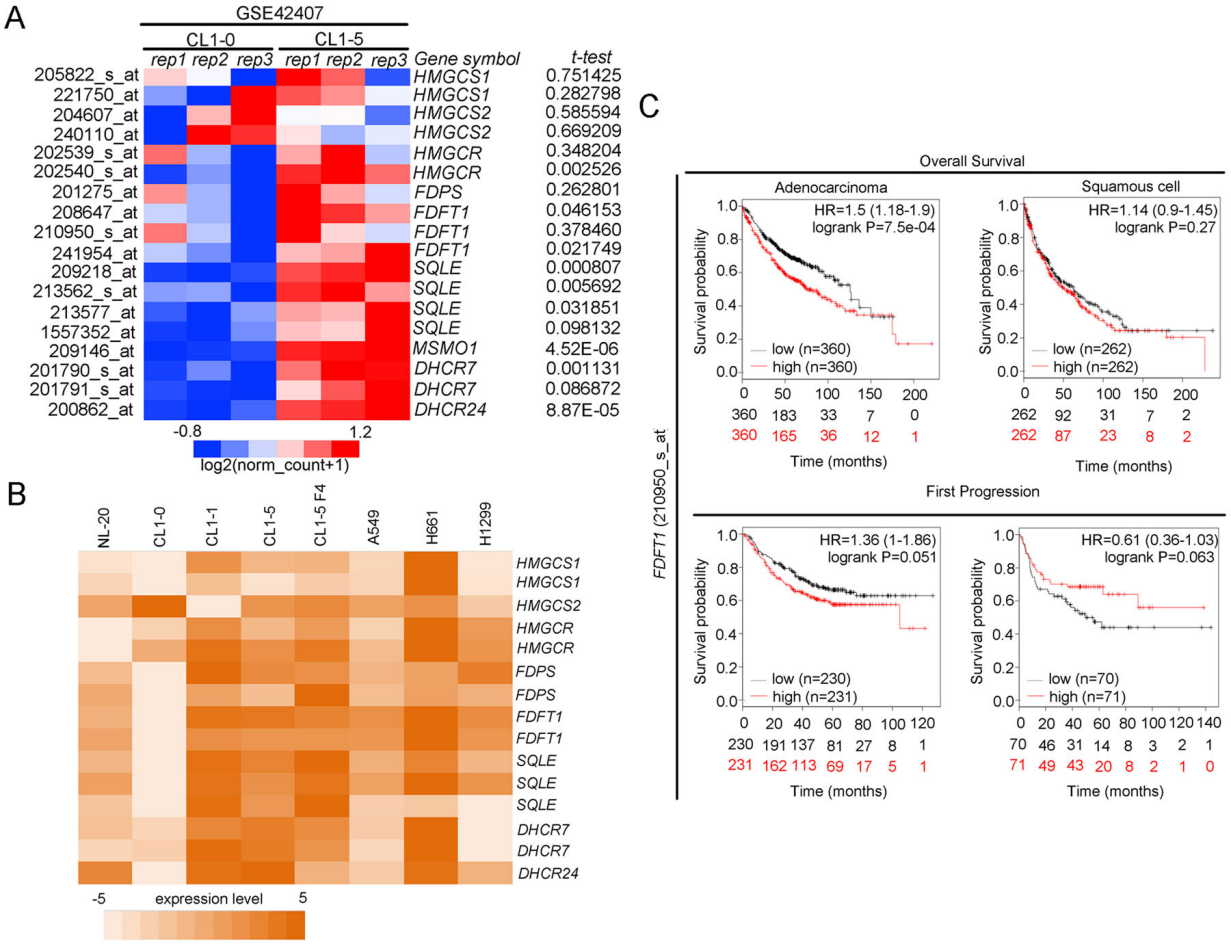
The cholesterol biosynthesis pathway is upregulated in highly invasive CL1-5 cells. **a** Expression of each enzyme involved in cholesterol biosynthesis in CL1-0/CL1-5 cells (NCBI/GEO/GSE42407). The significance was determined by Student’s *t* test. **b** Expression of each enzyme involved in cholesterol synthesis in various lung cancer cell lines (NCBI/GEO/GSE7670). **c** The *FDFT1* (210950_s_at, SQS) expression level is correlated with the survival curve in patients with lung adenocarcinoma and squamous cell carcinoma subtypes.

In our study, we aimed to identify a key metabolic pathway that is aberrantly overexpressed in invasive lung cancer cell and investigate its role. CL1-1, CL1-2, CL1-3, CL1-4, and CL1-5 were a series of lung cancer cell lines derived from CL1-0 cells and selected by Transwell that had increased invasiveness^16^. In our previous report, we analyzed a gene signature (enzyme annotation) that had differential expression in CL1-0 and CL1-5 (GSE7670)^7^. We identified that the cholesterol biosynthesis pathway was upregulated in highly invasive CL1-5 lung cancer cells. The cholesterol biosynthesis pathway has eight enzymes that were upregulated in CL1-5 cells, including (1) hydroxymethylglutaryl-CoA synthase 1 (*HMGCS1*), (2) farnesyl-diphosphate synthase (*FDPS*), (3) farnesyl-diphosphate farnesyltransferase (*FDFT1*, also known as squalene synthase, SQS), (4) squalene epoxidase (*SQLE*); (5) methylsterol monooxygenase 1 (MSMO1), (6) cytochrome P450, family 51, subfamily A (*CYP51A1*); (7) 7-dehydrocholesterol reductase (*DHCR7*), and (8) 24-dehydrocholesterol reductase (*DHCR24*) (Fig. S1)^7^. Moreover, we evaluated the expression levels of each cholesterol-related enzyme in CL1-0 and CL1-5 cells through a microarray dataset (GSE42407). Based on the results of a statistical analysis of three replicate samples and *P*-values, the expression of *HMGCR*, *FDFT1*, *SQLE*, *MSMO1*, *DHCR7*, and *DHCR24* were significantly different between CL1-0 and CL1-5 cells (Fig. 1a). We further verified the expression levels of each enzyme in a lung cancer cell panel. The heat map shows upregulation of *FDFT1 (SQS)* in several cell lines compared with the benign and nontumorigenic bronchial epithelial NL-20 cells (Fig. 1b) (GSE7670). Moreover, we detected the expression of the *FDFT1* (SQS*)* gene in a clinical cohort using a specific probe (210950_s_at). A high level of *SQS* expression was correlated with the survival rate, including overall survival and survival to the first progression, particularly in patients with the lung adenocarcinoma subtype (Fig. 1c). SQS is the first enzyme of the cholesterol branch that is upregulated in highly invasive lung cancer cell lines. Overexpression of SQS promotes lung cancer invasion and metastasis in vitro and in vivo and enhances cholesterol biosynethsis^7^. Thus, SQS plays an important role in lung cancer progression.

### OPN is required for SQS-induced lung cancer cell migration/invasion

**Fig. 2:**
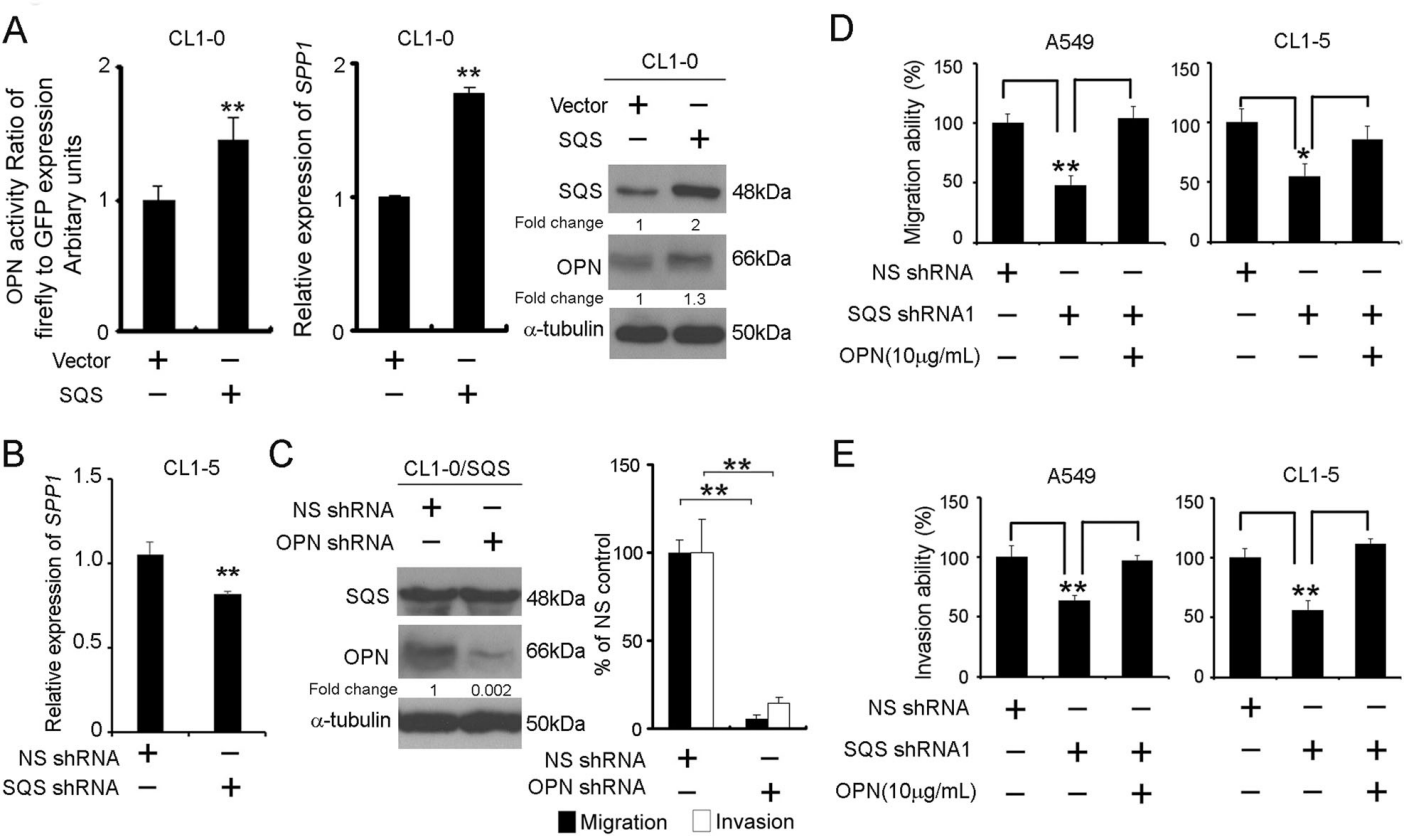
OPN mediates the migration and invasion of lung cancer cells overexpressing SQS. **a** *Left* panel, OPN promoter activity assay in CL1-0/SQS cells. CL1-0/vector cells were used as controls, and GFP served as a transfection internal control. After 48 h, green fluorescence was detected in the cells, followed by treatment with firefly luciferase for luminescence determination. *Middle* panel, Expression of *SPP1* (OPN) mRNA in CL1-0/SQS cells compared with CL1-0/Vector. *Right* panel, OPN protein levels in cells overexpressing SQS compared with the vector control. **b** Expression of the *SPP1* (OPN) mRNA in CL1-5/shSQS cells compared with CL1-5/nonsilencing (NS) control. Data are presented as the means ± SDs; **P < 0.01. The significance was determined by Student’s *t* test. **c** *Left* panel, western blot analysis of SQS and OPN levels in CL1-0/SQS cells after OPN knockdown. *Right* panel, Invasion and migration capabilities of CL1-0/SQS cells after OPN knockdown. **d**, **e** Effects of OPN replenishment on the migration/invasion of SQS-silenced A549 and CL1-5 cells. SQS-silenced cells were incubated in the presence or absence of OPN (10 μg/mL) and subjected to migration (**d**) and invasion (**e**) assays. Data are presented as the means ± SDs; *P < 0.05, **P < 0.01. The significance was determined by Student’s *t* test.

To identify the molecular mechanism and downstream pathways by which SQS regulates lung cancer cell progression, we analyzed the GSE37868 (as an mRNA microarray of CL1-0/SQS compared with CL1-0/Vector) datasets^7^, and secreted phosphoprotein 1 (*SPP1*; also known as osteopontin, OPN) was the top-ranking downstream factor that was upregulated upon SQS overexpression in CL1-0 cells. Therefore, we evaluated whether SQS regulated OPN expression in SQS-overexpressing cells. A promoter assay, real-time polymerase chain reaction and western blot analysis confirmed that OPN was upregulated in SQS-overexpressing CL1-0 cells compared with the vector control cells (Fig. 2a). Knockdown of SQS significantly decreased expression of OPN in CL1-5 (Fig. 2b). Overexpression of SQS was significantly increased migration and invasion capabilities in CL1-0 cells (Fig. S2A). We further investigate the effect of OPN on migration and invasion abilities of CL1-0/SQS cells. We observed a significant inhibition of migration and invasion following the knockdown of OPN with an shRNA in SQS-overexpressing cells (Fig. 2c and Fig. S2B). Conversely, the replenishment of OPN in SQS knockdown cells significantly restored the invasion and migration of A549 and CL1-5 cells (Fig. 2d, e and Fig. S2C, D).

### SQS induces metabolic reprogramming

**Fig. 3:**
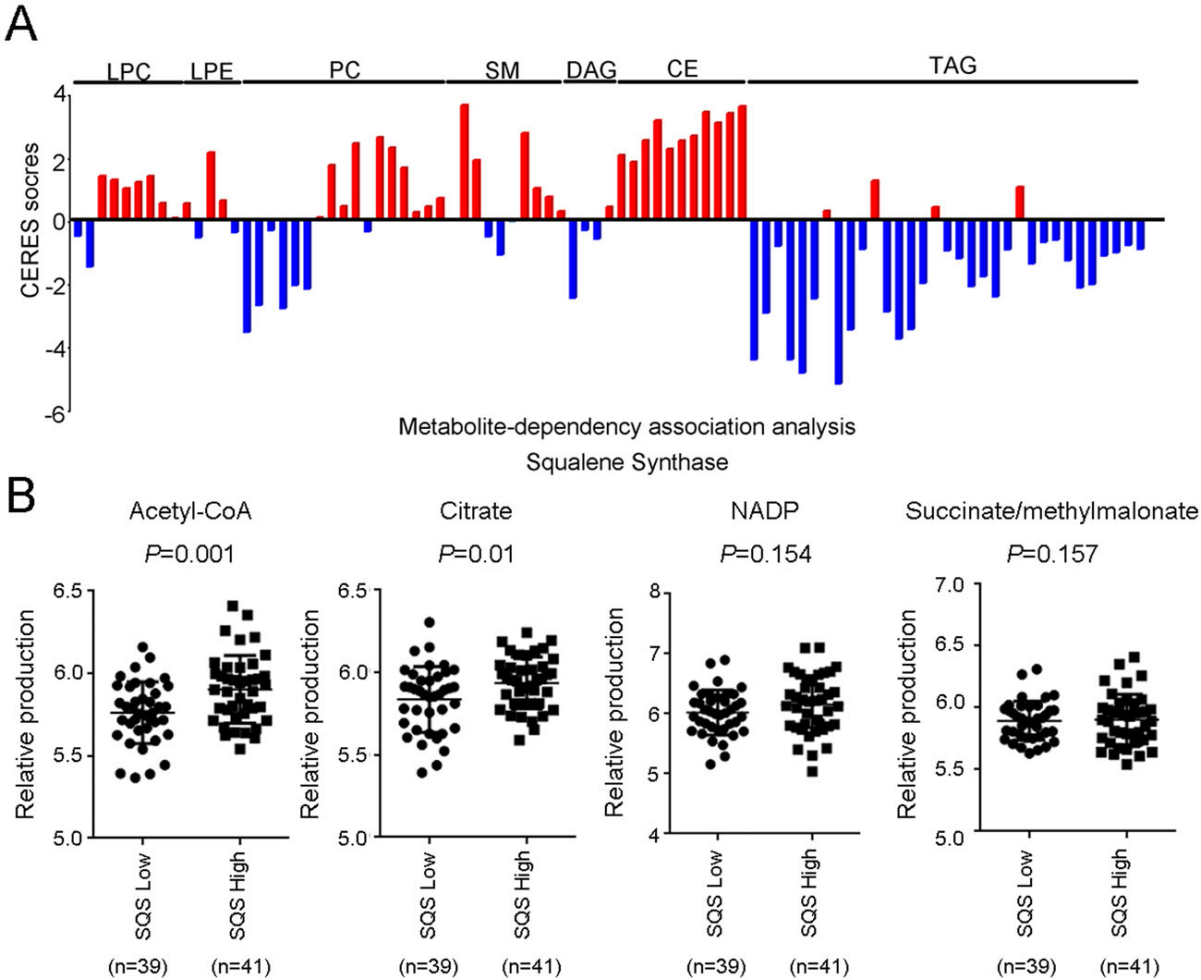
SQS expression was correlated with changes in metabolic signatures in lung cancer cell lines. **a** The metabolite-dependency association analysis shows several lipid products in cells with altered SQS expression. LPCs lysophophatidylcholines, LPEs lysophosphatidylethanolamines, PCs phosphatidylcholines, SMs sphingomeylins, DAGs diacylglycerols, CEs cholesteryl esters, TAGs triacylglycerols, CERES gene dependencies. **b** These swarm plots show several metabolites in lung cancer cell lines with altered SQS expression. The significance was determined by Student’s *t* test.

Knockdown of SQS reduced the cholesterol levels and inhibited invasion ability in lung cancer cell lines. Furthermore, knockdown of the downstream enzyme of SQS (SQLE, CYP51A1, MSMO1, and DHCR7) also significantly inhibited migration/invasion capabilities compared with the upstream enzyme (HMGCS1 and HMGCR2) in lung cancer cell lines^7^. We determined the metabolic signatures of lung cancer cells from the Cancer Cell Line Encyclopedia (CCLE) database to describe the metabolic events induced by SQS in lung cancer. Robin et al. developed the CERES score to summarize the gene-level dependency from CRISPR-Cas9 knockdown dataset across 342 cancer cell lines^17^. Furthermore, CERES is combined with the metabolic signature to define metabolite-dependent associated with gene levels in cancer cells^18^. Using the metabolite-dependency association algorithm, we noticed substantial changes in the levels multiple metabolites, particularly cholesteryl esters (CE) (Fig. 3a). Previous studies have defined rafts as cholesterol-rich microdomains in the plasma membrane that play a role in a number of signaling processes involving growth factor receptors, T-cell receptors, and the TNF receptor superfamily^19–23^. Silencing the TNF receptor inhibited migration/invasion capabilities in SQS-overexpressing lung cancer cells^7^. Moreover, we dissected the metabolite panels of various lung cancer cells. We analyzed the correlation between SQS expression and the ratio between glycolysis/OXPHOs in NSCLC cell lines (*n* = 80). We stratified cell lines into an SQS-low group (<70) and an SQS-high group (≧70) and observed SQS expression was significantly associated with acetyl-CoA (*P* = 0.001) and citrate (*P* = 0.01) levels (Fig. 3b and Appendix No. 1). Thus, SQS expression is correlated with metabolic signature changes in lung cancer cell lines.

### OPN modulates invasion through a cholesterol-dependent increase in ERK phosphorylation

**Fig. 4:**
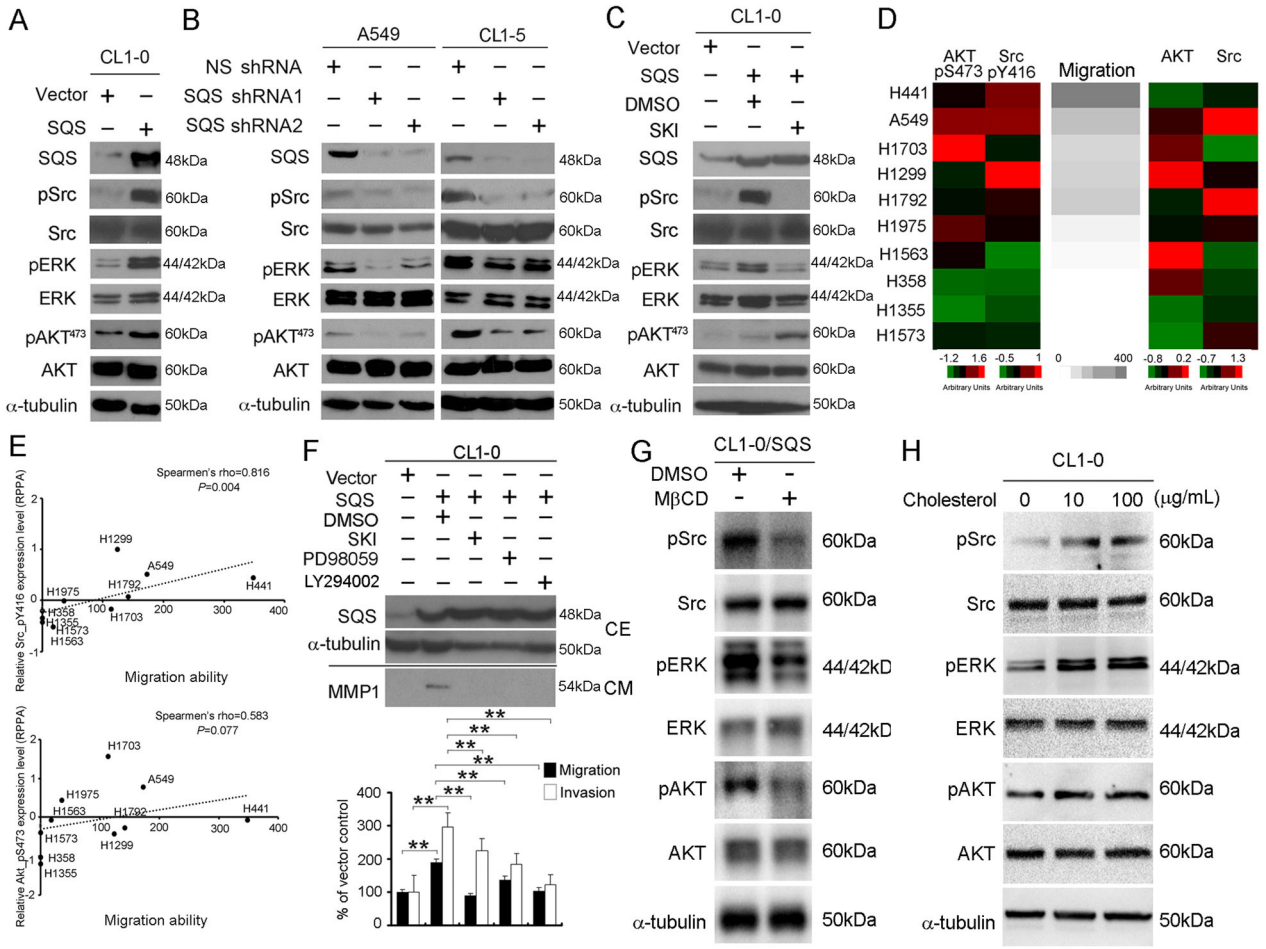
SQS activates the Src and ERK pathways in lung cancer cells. **a** CL1-0 cells infected with the vector and SQS overexpression plasmid were subjected to western blot analyses of SQS, pSrc, Src, pERK, ERK, pAKT^473^, AKT and α-tubulin levels. **b** Comparison of pSrc, Src, pERK, ERK, pAKT^473^, and AKT levels between SQS knockdown A549 and CL1-5 cells and nonsilenced shRNA control cells. **c** Western blots showing the levels of pSrc, Src, pERK, ERK, pAKT^473^, AKT and α-tubulin in CL1-0/SQS cells after treatment with Src kinase inhibitor-1 (SKI, 10 μM). **d** Heatmap showing the protein levels of AKT, AKT_pS473, Src, and Src_pY416 and migration ability in the lung cancer cell panel. **e** Plot showing the correlations between the protein levels of AKT_pS473 protein/Src_pY416 protein and migration in the lung cancer cell panel. **f** CL1-0/SQS cells were treated with or without DMSO, Src inhibitor-1 (SKI) (10 μM), PD98059 (20 μM) and LY294002 (10 μM). *Top* panel, The total protein samples were subjected to western blot analyses of SQS and α-tubulin levels (cell extract, CE). *Middle* panel, MMP1 protein expression levels from condition medium (CM). *Bottom* panel, Invasion (open) and migration (filled) of CL1-0/SQS cells treated with DMSO, SKI, PD98059 and LY294002 compared to CL1-0/Vector cells. Data are presented as the means ± SDs. **P < 0.01. **g** CL1-0/SQS cells were treated with or without MβCD (10 mM) for 30 min 37 °C. Western blot analyses of SQS, pSrc, pERK and pAKT and α-tubulin levels of CL1-0/SQS cells. **h** Effect of cholesterol replenishment on the phosphorylation of Src, ERK, and AKT in CL1-0 cells.

We postulated that the upregulation of SQS might induce an enrichment and activation of receptors in rafts. Previous studies have shown that Src family kinases (SFKs) are localized in the rafts^24^, and we evaluated the expression of Src pathway-related kinase in CL1-0/SQS cells by western blot analysis. Overexpression of SQS increased the phosphorylation of Src (Tyr416), ERK1/2, and AKT (Ser473), whereas SQS knockdown reduced the phosphorylation of Src, ERK1/2 and AKT in A549 and CL1-5 cells (Fig. 4a, b). Knockdown of OPN significantly decreased the expression of pSrc, pERK1/2, and pAKT in CL1-0/SQS cells (Fig. S3A). We focused on Src in the next experiment because OPN increases the phosphorylation of ERK1/2 and AKT via Src^13^. Similarly, the KEGG analyzed shows PI3K-AKT and MAPK signaling were upregulated in SQS overexpression cell by using DAVID software (GSE37868, Table S1). We next investigated whether SQS increased the phosphorylation of ERK1/2 and AKT via Src. As shown in Fig. 4c, SQS-induced phosphorylation of Src and ERK1/2 was abolished by an Src inhibitor (Src kinase inhibitor-1, SKI).

We analyzed the levels of proteins involved in these canonical pathways in lung cancer cells using a reverse-phase protein lysate microarray (RPPA) to confirm that SQS promotes lung cancer metastasis through the Src/ERK pathway. The phosphorylation of AKT (S473) and Src (Y416) strongly correlated with the migration ability of lung cancer cell lines (Fig. 4d). We further evaluated the correlations between pAKT and pSrc levels with the migration abilities of lung cancer cell lines. The correlation plots confirmed the positive correlations between pSrc levels with the migration abilities of a lung cancer cell panel (Spearman’s *rho* = 0.816, *P* = 0.004) (Fig. 4e). In addition, we also observed a significant inhibition of the migration and invasion of SQS-overexpressing cells following a treatment with the SKI (Src inhibitor), PD98059 (MEK/ERK inhibitor) and LY294002 (PI3K inhibitor) (Fig. 4f). We examined whether SQS mediated MMP1 expression through Src, ERK, and PI3K in CL1-0/SQS cells. Treatment with SKI, MEK, and PI3K inhibitor reduced the MMP1 expression and inhibited invasion/migration capabilities in CL1-0/SQS cells (Fig. 4f and Fig. S3B). Next, we investigated whether rafts were involved in the OPN/kinase axis in SQS-overexpressing cells. Treatment with methyl-β-cyclodextrin (MβCD, as a detergent that cholesterol depletes in the rafts), reduced the expression of pSrc, pERK1/2, and pAKT in CL1-0/SQS cells (Fig. 4g). Conversely, cholesterol treatment increased the phosphorylation of Src, ERK1/2, and AKT in CL1-0 cells that typically display a low level of invasion (Fig. 4h). Furthermore, we also observed that CD44 was not involved in the SQS/OPN pathway in SQS-overexpressing cells, in which the cell lines express different levels of CD44 (Fig. S3C, D). Taken together, SQS modulates OPN expression to increase the phosphorylation of Src, ERK1/2 and AKT, and subsequently promotes lung cancer metastasis.

### SQS upregulation correlates with OPN expression in lung cancer

**Fig. 5:**
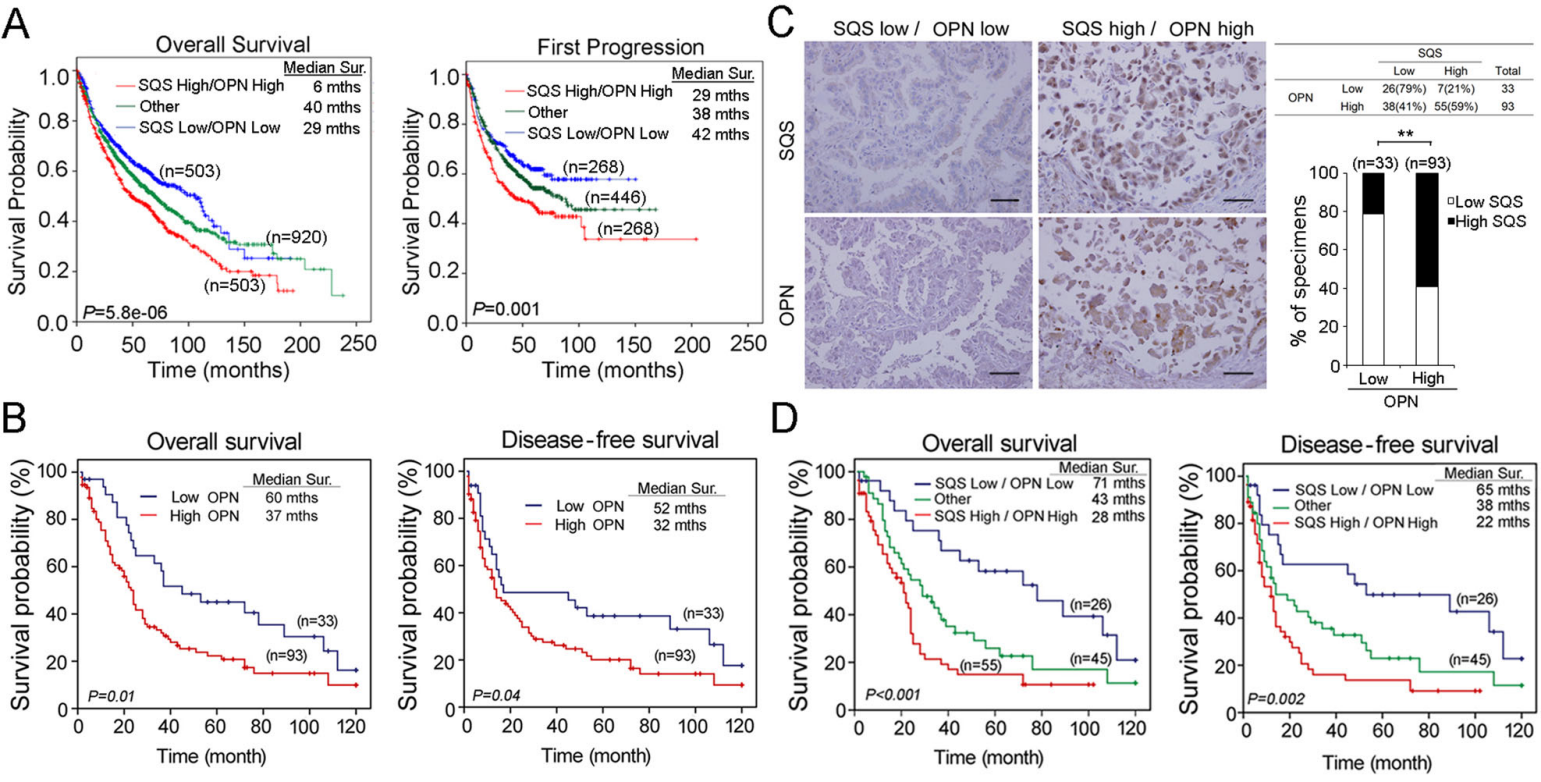
The expression of genes in the SQS/OPN axis is correlated with poor survival among patients with lung cancer. **a** Kaplan–Meier analysis of overall survival (left) and survival to the first progression (right) among patients with lung cancer is achieved by examining the combination of *SQS* and *OPN* mRNA expression levels. other: SQS low/OPN high and SQS high/OPN low. **b** Kaplan–Meier analysis of the overall survival (left) and disease-free survival (right) in 126 patients with lung cancer stratified according to SQS and OPN protein levels. **c** *Left*, Representative images of SQS and OPN IHC staining in serial sections of lung cancer tissues derived from patients. *Right*, Quantification of SQS and OPN IHC staining in lung cancer tissues. *N* = number of specimens. **P, < 0.01. Scale bar: 100 μm. **d** Kaplan–Meier analysis of overall survival (left) and disease-free survival (right) in 126 patients with lung cancer stratified according to the SQS and OPN protein levels.

We assessed the expression of SQS/OPN in patients with lung cancer using publicly available cDNA microarray datasets and online survival analysis software to evaluate the ability of the SQS/OPN axis to serve as an independent prognostic factor for patients with lung cancer^25^. *SPP1* (OPN) was upregulated in lung tumors and associated with a poor overall survival and survival to the first progression (Fig. S4A, B). We further confirmed that the expression of the *SQS* with *OPN* mRNAs combined in the clinical cohort exerted a synergistic effect compared with the expression of the *OPN* mRNA alone. We downloaded the expression levels of *SQS* and *OPN* from the KM plotter website^25^. This metacohort contains several microarray datasets (GSE series), CAARRAY and TCGA. In the clinical part, the website collected the survival time of overall survival (OS) and first progression (FP) of each patient. They further defined the low and high expression levels of *SQS* and *OPN*, respectively (0 = low, 1 = high). Therefore, we merged the *SQS* and *OPN* to redefine high (2), others (1) and low (0) to redraw the survival curve by Kaplan–Meier analysis. *SQS* High/*OPN* High represented a shorter OS and FP survival compared with *SQS* Low/*OPN* Low in patients with lung cancer (Fig. 5a). Moreover, we examined OPN protein expression in lung cancer tissues using immunohistochemical (IHC) staining and correlated OPN expression with the clinicopathological factors of patients with lung cancer. OPN expression was scored according to intensity on a scale ranging from 0-3 points. Using the defined scoring criteria, we stratified scores of 0 and 1 as low OPN expression, and scores of 2 and 3 were categorized as high OPN expression. High OPN expression levels were associated with smoking (*P* = 0.006), the histological type (*P* = 0.014), an advanced stage (stage III-IV, *P* = 0.005) and regional lymph node metastasis (*P* = 0.024) (Table S2 and Fig. S5). Moreover, the survival analysis revealed a significant correlation between high OPN levels and a poor overall survival (OS) and disease-free survival (DFS) compared with low OPN levels (Fig. 5b). The univariate analyses of OS and DFS revealed that OPN expression was a significant predictor of survival (Table S3).

**Fig. 6:**
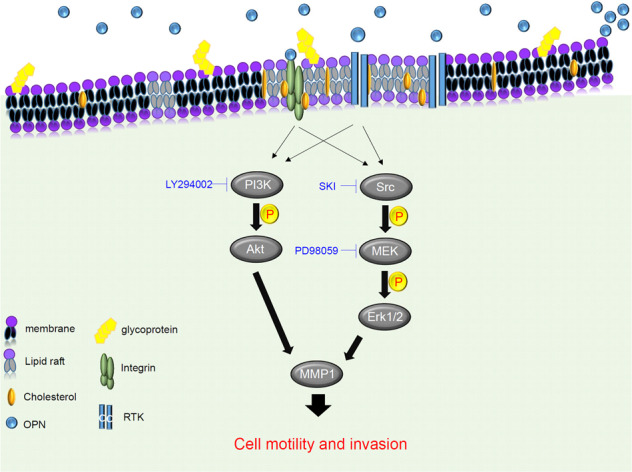
Model of the role of the SQS-Src/ERK-OPN axis in lung cancer. SQS promotes cholesterol production and enhances lung cancer motility and invasion through the OPN axis. OPN activates ERK phosphorylation status through a cholesterol-dependent pathway.

We next investigated whether SQS expression correlated with OPN expression in patients with lung cancer using an IHC analysis and identified a significant positive correlation between SQS and OPN expression (Fig. 5c, Spearman’s nonparametric correlation test, correlation coefficient = 0.334, *P* < 0.001, Table S4). In the analysis of the SQS high/OPN high cohort compared with the SQS low/OPN low cohort, the cumulative survival analysis showed a significant decrease in survival in the high cohort (Fig. 5d). These data suggest that upregulated SQS enhances invasion by upregulated OPN, leading to phosphorylation Src, ERK and AKT, and increased MMP1 expression (Fig. 6).

## Discussion

Here, SQS overexpression was associated with a concurrent upregulation of OPN. SQS promotes the invasion and migration of lung cancer cell lines through OPN. According to the results of the clinicopathological analysis, the upregulation of OPN was associated with smoking, histological stage and lymph node metastasis in patients with lung cancer. Moreover, patients with lung cancer presenting with high OPN expression displayed shorter overall survival and disease-free survival (Fig. 5b). SQS induced the phosphorylation of Src, ERK1/2, and AKT in lung cancer cell lines, but the levels of pSrc, pERK1/2, and MMP1 were decreased after treatment with SKI (Src inhibitor) (Fig. 4c, f). MβCD treatment inhibited the phosphorylation of Src, ERK1/2 and AKT in CL1-0/SQS cells. These findings suggest that SQS promoted invasion/migration abilities via the OPN pathway in lung cancer cells.

OPN is highly expressed in lung antigen-presenting cells (APCs) of mice (smoke-exposed) and is required for the production of IL-17A and smoke-induced emphysema through inhibiting the expression of transcription factor IFN regulator 7(*Irf*7)^26^. Moreover, IL-17A regulates tumor growth in lung cancer^27^ and emphysema in patients with chronic obstructive pulmonary disease (COPD), which is related to lung cancer development^28^. Overexpression of OPN has been reported to be associated with poor survival of patients with lung cancer^29^. The prognostic significance of circulating OPN levels has been reported in patients with lung cancer, in whom elevated plasma OPN levels are associated with disease recurrence after tumor resection^30^. In addition, TNF-α-induced OPN expression in HN-22 cells growing on fibronectin supports occurs via β1 integrin and ERK^31^; in the present study, OPN was upregulated in SQS-overexpressing cells and correlated with smoking status in lung cancer patients (Fig. 2a and Table S2). In our previous reports, we found that SQS facilitates TNFR1 enrichment into rafts to enhance lung cancer migration/invasion through the NF-κB-MMP1 axis^11^. In this study, we discovered other features of SQS. The production of ceramide (CE) and phosphatidylcholine (PC) were upregulated in high SQS-expressing cell lines (Fig. 3a). SQS gene is not only involved in raft remodeling but also regulates the cellular microenvironment.

OPN binds to integrins and CD44 and contains a protease-hypersensitive site^32^. OPN interacts with integrins to induce the activation of phosphoinositide 3-kinase (PI-3Kinase), mitogen-activated protein kinase (MAPK), phospholipase C-γ and protein kinase C through the c-Src-dependent transactivation of the epidermal growth factor receptor (EGFR) in cancer cells^33,34^. Furthermore, targeting ERK with U0126 (ERK1/2-specific MEK1/2 inhibitor) reduced OPN expression in MC3T3-E1 cells but not p38 and JNK kinase^35^. Mouse OPN promoter was stimulated by v-Src in HT1080 cells^36^. However, OPN expression did not change with LY294002 (PI3K inhibitor) treatment in MHCC-97L cells^37^. In the present study, SQS overexpression increased OPN, pSrc, pERK1/2 and pAKT^473^ levels in lung cancer cells, whereas knockdown of SQS or treatment with SKI significantly reduced the levels of pSrc and pERK1/2 (Fig. 4a–c). Similar to our results, Chitose et al. showed that Src activation was limited by cholesterol-enriched raft microdomains^38^. In recent years, Holzer et al. mentioned that Src is necessary for JNK activation to catalyze saturated fatty acids^39^. Saturated fatty acid conditions include type II diabetes and insulin resistance. In several cancer types, fatty acids are also increased through metabolic reprogramming. In addition, it has been claimed that the aggregation of rafts is activated by Src in several cancer types^40,41^. In this manuscript, we provide many clues consistent with these references. These views reflect the importance of rafts and are related to typical oncogenic pathways.

Previous studies have shown suppression of Src family kinase blocks epidermal growth factor-induced phosphorylation of AKT in T47D breast cancer cells. However, Src inhibitor (PP1) inhibits EGF-induced phosphorylation of AKT in T47D cells at 15 min, and pAKT recovered after treatment for 30 min^42^. In our studies, we treated Src inhibitors in CL1-0/SQS cell for 16 h (Fig. 4c). The ending point was referring to the MMP1 study by Yukihiro Hojo et al.^43^. Therefore, we speculate that pAKT has resumed its expression after a long period of drug treatment. Expression of pAKT was decreased after treatment with MβCD in CL1-0/SQS cells (Fig. 4g). Furthermore, *SQS* mRNA levels were significantly upregulated in lung cancer patients with *KRAS* mutations^7^. Ras has also been observed to activate and interact with the catalytic subunit of type I phosphatidylinositol-3-kinase (PI3Ks), leading to PI3K translocation to the cell membrane and conformational changes by activation of lipid kinase^44,45^. This is the first study showing that overexpression of SQS increased the expression of pSrc, pERK and pAKT in CL1-0 cells. However, the detailed mechanism by which SQS promotes the expression of pAKT is worthy of further investigation.

Knockdown of SQS destroyed rafts in lung cancer cell lines^7^. CD44 shedding and the delocalization of focal adhesion complexes from rafts induced by cholesterol depletion inhibited tumor cell migration^46,47^. The OPN-CD44 interaction contributes to the survival of BA/F3 cells by activating PI3K-AKT signaling^48^. Exogenous OPN treatment increases CD44 expression and osteoclast motility in OPN^−/−^ osteoclasts^49^. OPN is upregulated in patients with lung cancer^50^ and interacts with splice variants of CD44 (CD44v) to promote the enrichment of CD44 in rafts, leading to increased survival rates in human cancer cell lines^51,52^. Here, we showed that overexpression of SQS increased pERK and pAKT in CL1-0 and H1355 cells, which have different levels of CD44 (Fig. 4a, Fig. S3C, D). These data suggested that CD44 was not involved in the SQS/OPN pathway. Cholesterol depletion leads to reduced phosphorylation of Src, ERK1/2, and AKT in CL1-0/SQS cells (Fig. 4g). Conversely, cholesterol replenishment induced the phosphorylation of Src, ERK1/2 and AKT in CL1-0 cells (Fig. 4h). Although we were unable to exclude the involvement of other pathways in Src phosphorylation, this study is the first to show that SQS overexpression increased pERK levels through Src.

Accumulating evidence has revealed a function for OPN in modulating tumor metastasis^53,54^. Recent reports have indicated that OPN activity is regulated by HIF-1α, is PI3K/AKT pathway-dependent and also accompanies EMT^55,56^. Moreover, OPN can directly interact with various membrane receptors, including EGFR, CD44, VEGF and integrins, thereby promoting cancer metastasis^57,58^. In our study, OPN knockdown significantly inhibited the migration and invasion of CL1-0/SQS cells, whereas OPN replenishment restored the migration and invasion of A549/shSQS and CL1-5/shSQS cells (Fig. 2c–e). According to a previous study, OPN induced MMP1 expression in smooth muscle cells through integrin αvβ3^59^. In our study, the migration and invasion of SQS-overexpressing cells were inhibited after treatment with Src (SKI), MEK (PD98059), and PI3K (LY294002) inhibitors. The SKI, MEK, and PI3K inhibitors also reduced MMP1 expression in SQS-overexpressing cells (Fig. 4f and Fig. S3B). Based on these findings, we surmised that SQS is required for OPN expression and subsequently promotes the migration and invasion of lung cancer. In this manuscript, we describe this new mechanism and the clinical outcomes of alterations in the SQS/OPN axis in lung cancer. Moreover, we screened these combined approaches in various cancer cell lines. As shown in the survival plots, breast, ovarian and gastric cancers exhibited similar trends (Fig. S6). In our future prospective study, we will develop and implement these strategies for modulating the SQS/OPN axis and expand them to multiple cancer types. We will then perform a clinical study to analyze the correlations with clinicopathological factors. In addition, we will consider combining options as a powerful therapeutic strategy or a prognostic/diagnostic approach for patients with cancer.

In conclusion, this study is the first to investigate the mechanism by which SQS promotes the migration and invasion capabilities of CL1-0 lung cancer cells through the OPN-Src-ERK pathway and its implications for the clinical setting. SQS expression was correlated with reduced overall survival and first progression survival, indicating that SQS is an independent prognostic factor. The combined expression of SQS and OPN could provide a prognostic biomarker for lung cancer metastasis.

## Materials and methods

### Cell lines and cell culture conditions

The cell lines CL1-0/Vector, CL1-0/SQS, CL1-5-nonsilence, CL1-5-shSQS-1 and CL1-5-shSQS-2 were maintained in RPMI supplemented with 10% FBS and 1% penicillin-streptomycin-glutamine (PSG). A549-nonsilence, A549-shSQS-1 and A549-shSQS-2 cells were maintained in DMEM containing 10% FBS and 1% PSG. SQS overexpression and knockdown were performed as described previously^7^. The SQS overexpression system was a pLenti-6.3/V5-DEST^TM^ vector (#V53306, ThermoFish), and the knockdown system was a pGIPZ shRNA vector (Open Biosystems) or pLKO.1 TRC cloning vector (National RNAi Core Facility Platform). The shRNA sequences are shown in Supplementary Table S5. Src Inhibitor-1 (SKI) (Cat. S2075, 10 μM), PD-98059 (a mitogen-activated protein (MAP) kinase inhibitor, Cat. P215, 20 μM) and cholesterol (Cat.C3045) were purchased from Sigma, St. Louis, MO, USA. All inhibitors were dissolved in DMSO. Cholesterol was dissolved in ethanol.

### Western blot analyses

Western blot analyses were performed using a previously method^7^. The antibodies were directed against SQS (1:2000, GTX104091, Genetex), OPN (1:1000, #18625, IBL), pSrc (1:1000, #2101, Cell Signaling Technology), Src (1:1000, #2109, Cell Signaling Technology), pERK (1:1000, #9101, Cell Signaling Technology), ERK (1:1000, #9102, Cell Signaling Technology), pAKT (1:1000, #4060, Cell Signaling Technology), AKT (1:1000, #4691, Cell Signaling Technology), MMP1 (1:1000, #10371-2-AP, Proteintech) and α-tubulin (1:10,000, #T5168, Sigma-Aldrich) (Supplementary Table S5). All western blot data are representative of at least three independent replicates.

### Promoter assay

For the OPN promoter assay, the pGL3-Basic-OPN (containing a fragment of the OPN promoter from bases −2267 to −1) plasmid used to detect the transcription of OPN was kindly provided by Dr. Pei-Jung Lu^60^. The pGL3-Basic plasmid served as a control, and pZsGreen N1 was used as an internal control for OPN transcription and transfection efficiency. Cells (5000 cells/well) were seeded in 96-well plates and incubated at 37 °C with 5% CO_2_, overnight. The CL1-0/Vector and CL1-0/SQS cells were transfected with 150 ng pGL3-Basic-OPN and 15 ng pZsGreen N1 by using X-tremeGENE HP DNA transfection reagent (Roche). After 48 h, the green fluorescence (as transfection control) and luciferase signals were measured by using a plate reader (Victor3, PerkinElmer) and ONE-Glo Luciferase assay (Promega, #E6120).

### Quantitative real-time PCR

Total RNA was extracted using TRIzol^M^ reagent (#15596026, Thermo Fisher), and cDNAs were synthesized using the SuperScript™ IV First-Strand Synthesis System (#18091200, Thermo Fisher). Quantitative real-time PCR was performed using the SYBR Green PCR Supermix (Bio-Rad). The primer sequences are shown in Supplementary Table S5.

### Cell migration/invasion assay and OPN replenishment

The invasion and migration assays were performed as previously described^7^. For OPN replenishment, cells were resuspended in serum-free medium containing OPN (10 μg/mL, #120-35, PEPROTECH, Rocky Hill, NJ) and added to the upper compartment of each well. All experiments were performed in quadruplicate.

### Conditioned medium collection

The cells were plated on 6-cm culture plates at a density of 1 × 10^6^ cells in complete medium, incubated for 24 h, and then washed twice with serum-free medium. For the collection of conditioned medium, cells were incubated with or without different inhibitors in 2 mL of serum-free medium for 24 h. Next, the medium was collected and concentrated^61^.

### Cholesterol depletion

CL1-0/SQS cells were plated at a density of 2 × 10^6^ cells in 10-dish plates in 10% FBS complete medium for 24 h at 37 °C. After 24 h, the CL1-0/SQS cells were washed twice with 1% FBS medium and then treated with or without MβCD (10 mM) for 30 min at 37 °C.

### In silico study

The clinical information and genomics matrix file of The Cancer Genome Atlas (TCGA) database (https://xenabrowser.net/heatmap/) were download from the USCS Xena browser website. All Gene Expression Omnibus Series (GSE) series datasets downloaded from Gene Express Omnibus (GEO) website were normalized and analyzed using GeneSpring software (Version 13.1.1., Agilent, Santa Clara, CA, USA) and the Kaplan–Meier plotter website (https://kmplot.com/analysis/). All of the data we downloaded from the browser included clinical parameters and expression levels of target genes in patients with lung cancer. This website applied microarray analysis or next-generation sequencing of each probe after normalization. High expression is defined as expression above the median level. In addition, we removed several clinical cases that lacked the corresponding parameters. We further divided the samples into two subtypes of lung cancer: lung adenocarcinoma and lung squamous cell carcinoma.

### In silico datasets and biostatistical analysis

For Fig. 1a, we downloaded GSE42407 from the GEO website. After normalization, we output all probes and selected cholesterol-related genes for heatmap analysis. Moreover, we calculated the p-value of each probe in the CL1-0 and CL1-5 groups (*n* = 3) through Student’s *t* test. Similarly, we downloaded GSE7670 from the GEO website, retained the lung cancer cell panel, performed the same procedures as in Fig. 1a, and plotted the heatmap in Fig. 1b. From RPPA (reverse-phase protein arrays) profiles from CCLE (Cancer Cell Line Encyclopedia, CCLE_RPPA_20181003.csv, https://portals.broadinstitute.org/ccle/data), we picked target events including Akt, Akt_pS473, Src, and Src_pY416 to compare with our available migration ability through Boyden’s chamber. Combining all the evidence, we evaluated these data with heatmaps (Fig. 4d) and correlation plots (Fig. 4e).

### Metabolism analysis

In Fig. 3b, we analyzed the correlation between SQS expression and the ratio between glycolysis/OXPHOs in NSCLC cell lines (*n* = 80). We recorded the expression levels of FDFT1 (SQS) and several glycolysis/OXPHOs-related products, including α-ketoglutarate, AMP, citrate, isocitrate, DHAP/glyceraldehyde 3P, F1P/F6P/G1P/G6P, fumarate/maleate/alpha- ketoisovalerate, lactate, malate, NAP, NADP, oxalate, PEP and succinate/methylmalonate (Appendix No. 1).

### KEGG analysis

DAVID Functional Annotation Bioinformatics Microarray Analysis is a prediction software that can be used for transcriptome datasets^62^. We set the threshold to >1.5-fold change and exported these probe lists from GeneSpring software. We further converted the gene ID list into gene symbols on the DAVID website and then predicted its corresponding signaling pathways (KEGG-system) with gene ontology.

### Specimens

A total of 126 lung cancer tissue samples were collected from Kaohsiung Medical University Hospital (from 1991 to 2007) after obtaining IRB approval (KMUH-IRB-2011-0286). The histological diagnosis and grade of lung cancer were determined according to the WHO classification. The clinicopathological features, including the tumor size, local invasion, lymph node involvement, and distal metastases, were determined according to the AJCC TNM classification of lung cancer^3^. The follow-up duration was up to 200 months. The study employed a retrospective design, and only archived surgically removed tumor samples embedded in paraffin were used; thus, we did not obtain a registration number from the ClinicalTrials.gov website. The requirement for informed consent was waived by the Institutional Review Board of Kaohsiung Medical University Hospital of Taiwan.

### Immunohistochemistry

Immunohistochemical (IHC) staining was performed using the Ventana IHC staining system (Ventana, Tucson, AZ). IHC was performed using the primary antibodies against SQS (1:100, GTX104091, Genetex) and OPN (1:1000, ab69498, Abcam). The OPN score was based on the intensity of IHC staining: −, negative; 1+, 0–20% of tumor cell stained; 2+, 20–50% of tumor cell stained; and 3+, >50% of tumor cell stained.

### Statistical methods

Student’s *t* test was used to analyze the statistical significance of the results obtained from least three independent experiments, and the data are presented as the means ± SDs. Differences in the clinicopathological characteristics were determined using the chi-square test. Survival curves were analyzed using the log-rank test (generated using the Kaplan–Meier method). Univariate and multivariate models were established using a Cox proportional hazards regression analysis to evaluate the prognostic significance of various factors. All statistical tests were two-sided. *P* < 0.05 was considered significant. Statistical analyses were performed using SPSS (Statistical Package for the Social Sciences, version 19.0) software.

## Supplementary information

Supplemental Information

Appendix No.1
