# Supplementary material for: Squalene synthase promotes the invasion of lung cancer cells via the osteopontin/ERK pathway
Source: Oncogenesis. 2020 Aug 29;9(8):78. doi: 10.1038/s41389-020-00262-2 (PMC7456423; doi:10.1038/s41389-020-00262-2)
Supplement: Supplementary file 1 — Supplemental Information [file 41389_2020_262_MOESM1_ESM.docx]

**Supplemental Information**

**Squalene Synthase Promotes the Invasion of Lung Cancer Cells via the Osteopontin/ERK Pathway**

Yi-Fang Yang^1^, Yu-Chan Chang^2,3*^, Yi-Hua Jan^3^, Chih-Jen Yang^4,5,6^, Ming-Shyan Huang^7^ and Michael Hsiao^3, 8*^

^1^ Department of Medical Education and Research, Kaohsiung Veterans General Hospital, Kaohsiung, Taiwan

^2^ Department of Biomedical Imaging and Radiological Sciences, National Yang-Ming University, Taipei, Taiwan

^3^ Genomics Research Center, Academia Sinica, Taipei, Taiwan

^4^ Division of Pulmonary and Critical Care Medicine, Department of Internal Medicine, Kaohsiung Medical University Hospital, Kaohsiung Medical University, Kaohsiung, Taiwan

^5^ Department of Internal Medicine, Kaohsiung Municipal Ta-Tung Hospital, Kaohsiung Medical University, Kaohsiung, Taiwan

^6^ Department of Respiratory Therapy, College of Medicine, Kaohsiung Medical University, Kaohsiung, Taiwan

^7^ Department of Internal Medicine E-DA Cancer Hospital School of Medicine

I-Shou University Kaohsiung, Taiwan

^8^ Department of Biochemistry, College of Medicine, Kaohsiung Medical University, Kaohsiung, Taiwan.

Correspondence to: Dr. Michael Hsiao, Genomics Research Center, Academic Sinica, 128 Academia Road, Section 2, Taipei 115, Taiwan. Phone: 886-2-2787-1243; Fax: 886-2-2789-9931; E-mail: [mhsiao@gate.sinica.edu.tw](mailto:mhsiao@gate.sinica.edu.tw)

or to Dr. Yu-Chan Chang, Department of Biomedical Imaging and Radiological Sciences, National Yang-Ming University, Taipei, Taiwan; E-mail: [jameskobe0@gmail.com](mailto:jameskobe0@gmail.com)

Inventory of all Supplemental Information

Supplemental Data

Figure S1 Related to Figure 1

Figure S2 Related to Figure 2

Figure S3 Related to Figure 4

Figure S4 Related to Figure 5

Figure S5 Related to Figure 5

Figure S6

Supplementary Tables 1-5

Supplementary Table 1 Related to Figure 4

Supplementary Table 2 Related to Figure 5

Supplementary Table 3 Related to Figure 5

Supplementary Table 4 Related to Figure 5

Supplementary Table 5 Related to KEY RESOURCES

**Supplementary Figure Legends**

**
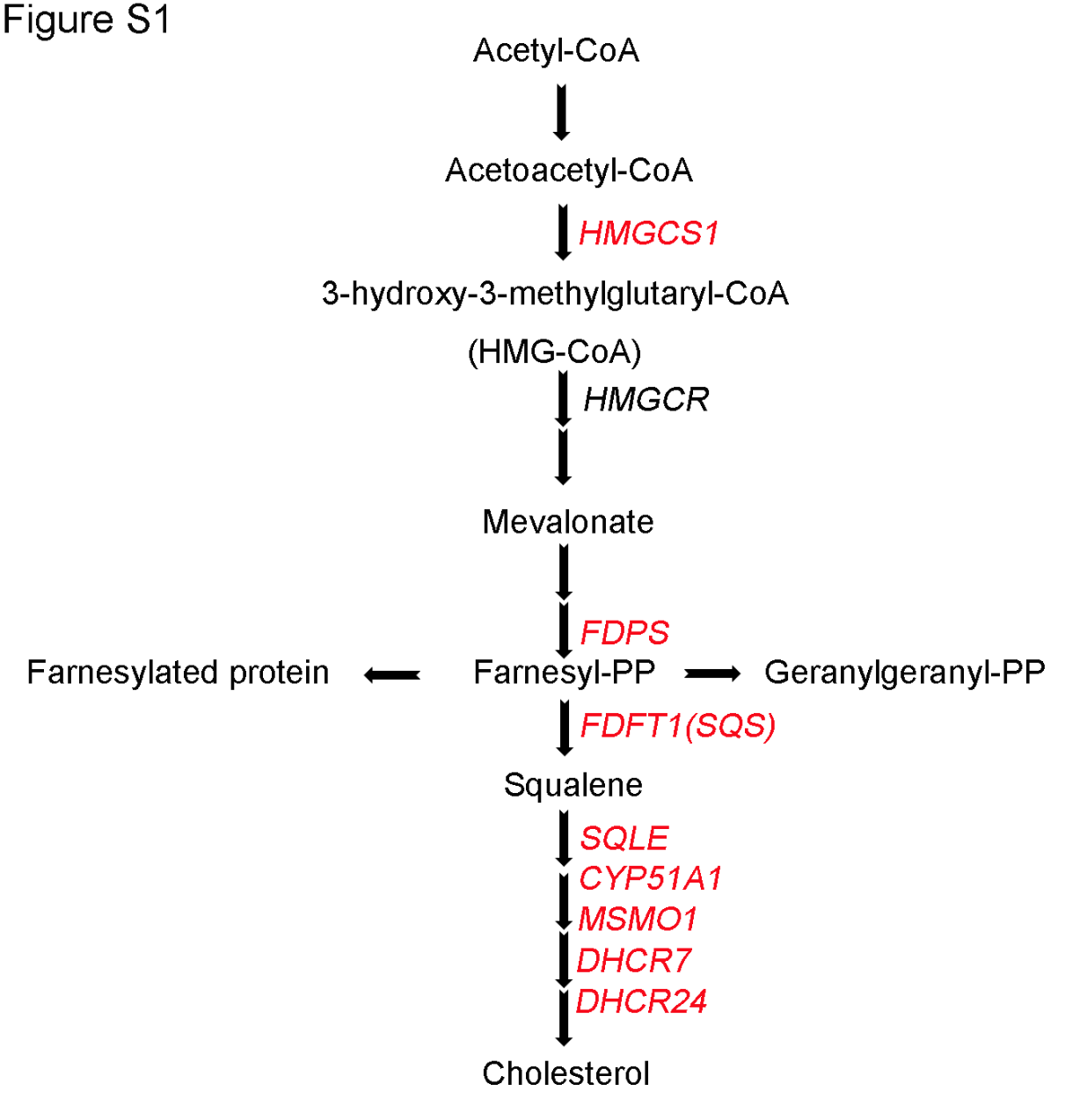
**

**Figure S1. The cholesterol biosynthesis pathway was upregulated in CL1-5 lung cancer cells.** Red shows upregulation in highly invasive CL1-5 cells compared with noninvasive CL1-0 cells (GSE7670).


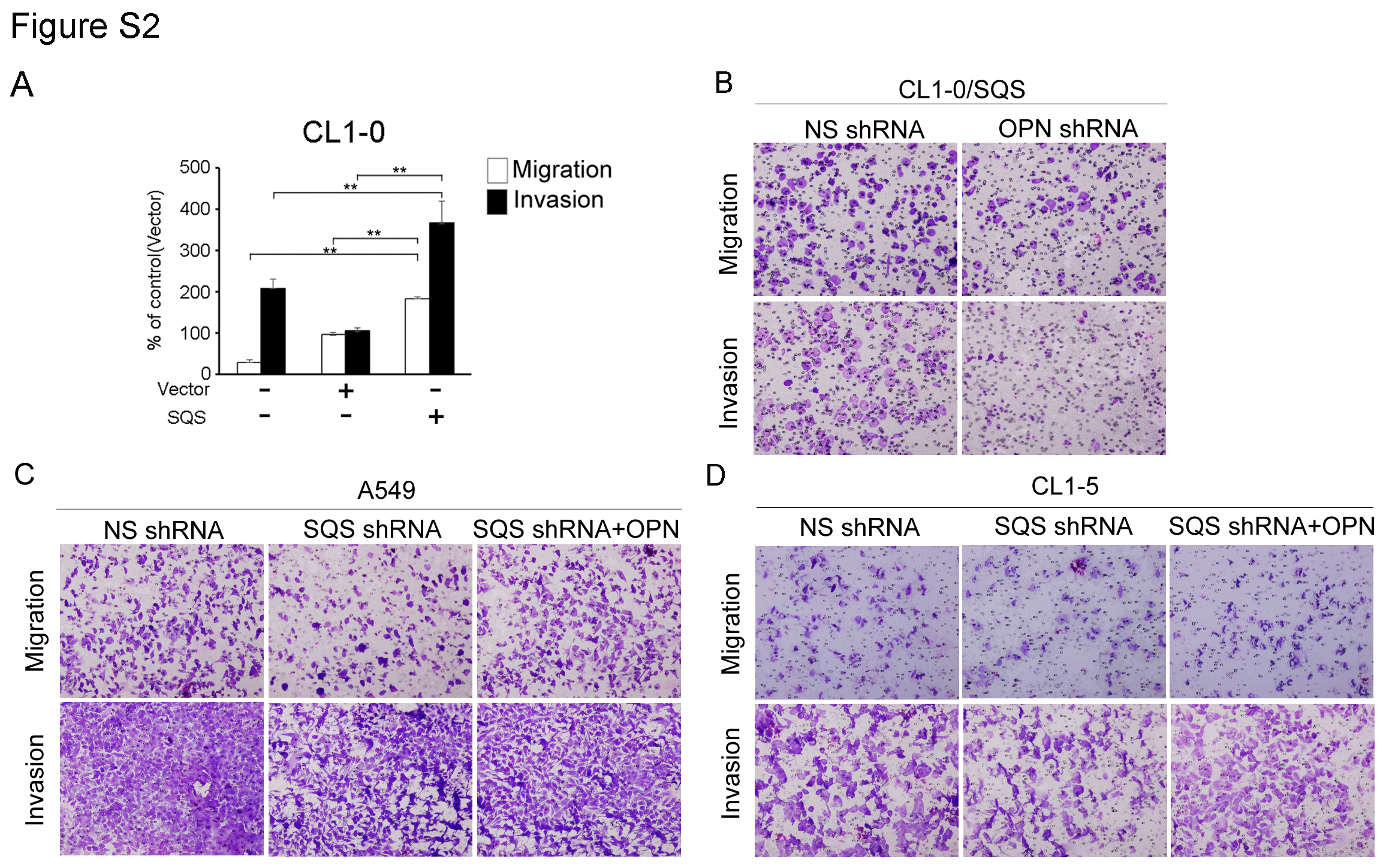


**Figure S2**. **Representative images showing the migration/invasion abilities of several cell models.** (A) Migration and invasion abilities of CL1-0 cells after vector and SQS expression. Data are presented as the means ± SDs; ***P*<0.01. The significance was determined by Student’s *t*-test. (B-D) Representative image of the invasion/migration capabilities of CL1-0/SQS after OPN knockdown (B), A549/shSQS (C) and CL1-5/shSQS (D) replenishment with OPN.





**Figure S3.** **SQS modulated MMP1 expression by the Src, ERK and AKT pathway in CL1-0/SQS cells**. (A) Expression of pSrc, pERK and pAKT in CL1-0/SQS cells after knockdown of OPN. (B) Expression of *MMP1* mRNA in CL1-0/SQS cells compared with vector. Reverse transcription polymerase chain reaction (RT-PCR) analysis of *SQS* and *MMP1* expression in CL1-0/SQS cells after treated with or without DMSO, Src inhibitor-1 (SKI) (10 μM), PD98059 (20 μM) or LY294002 (10 μM). (C) Expression of SQS, CD44 and α-tubulin in CL1-0/SQS cells. (D) Left, Expression of SQS, OPN, CD44 and α-tubulin in H1355/SQS cells. Right, Expression of pERK and pAKT in H1355/SQS cells.

**
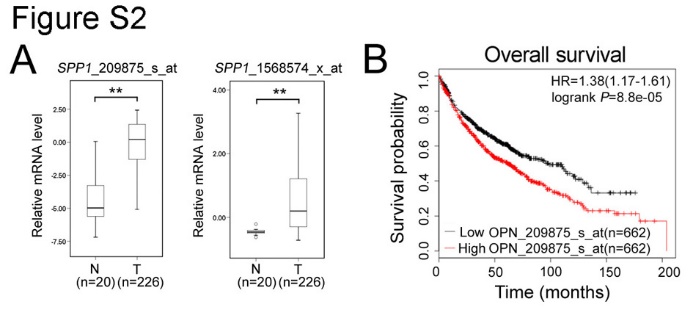
**

**Figure S4**. ***SPP1* (OPN) expression levels are correlated with survival among patients with lung cancer.** (A) *SPP1* expression in lung cancer tissues (adenocarcinoma, NCBI/GEO/GSE31210). Data are presented as the means ± SDs; ***P* < 0.01. (B) Kaplan-Meier graph of survival to the first progression among patients included in publicly available lung cancer microarray datasets and stratified according to *OPN* (SPP1) expression.


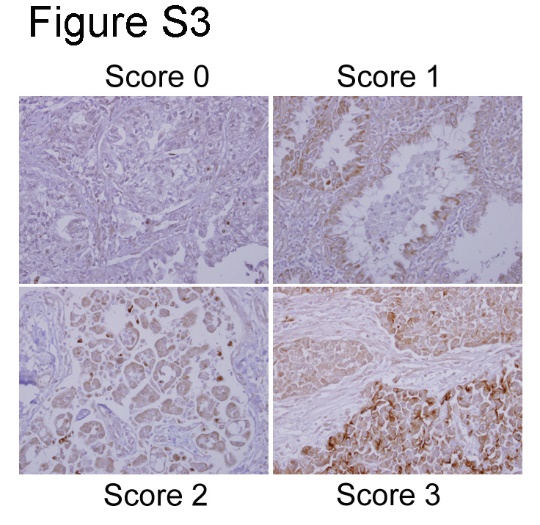


**Figure S5**. **IHC scoring of the OPN protein in the lung cancer tissue array.**


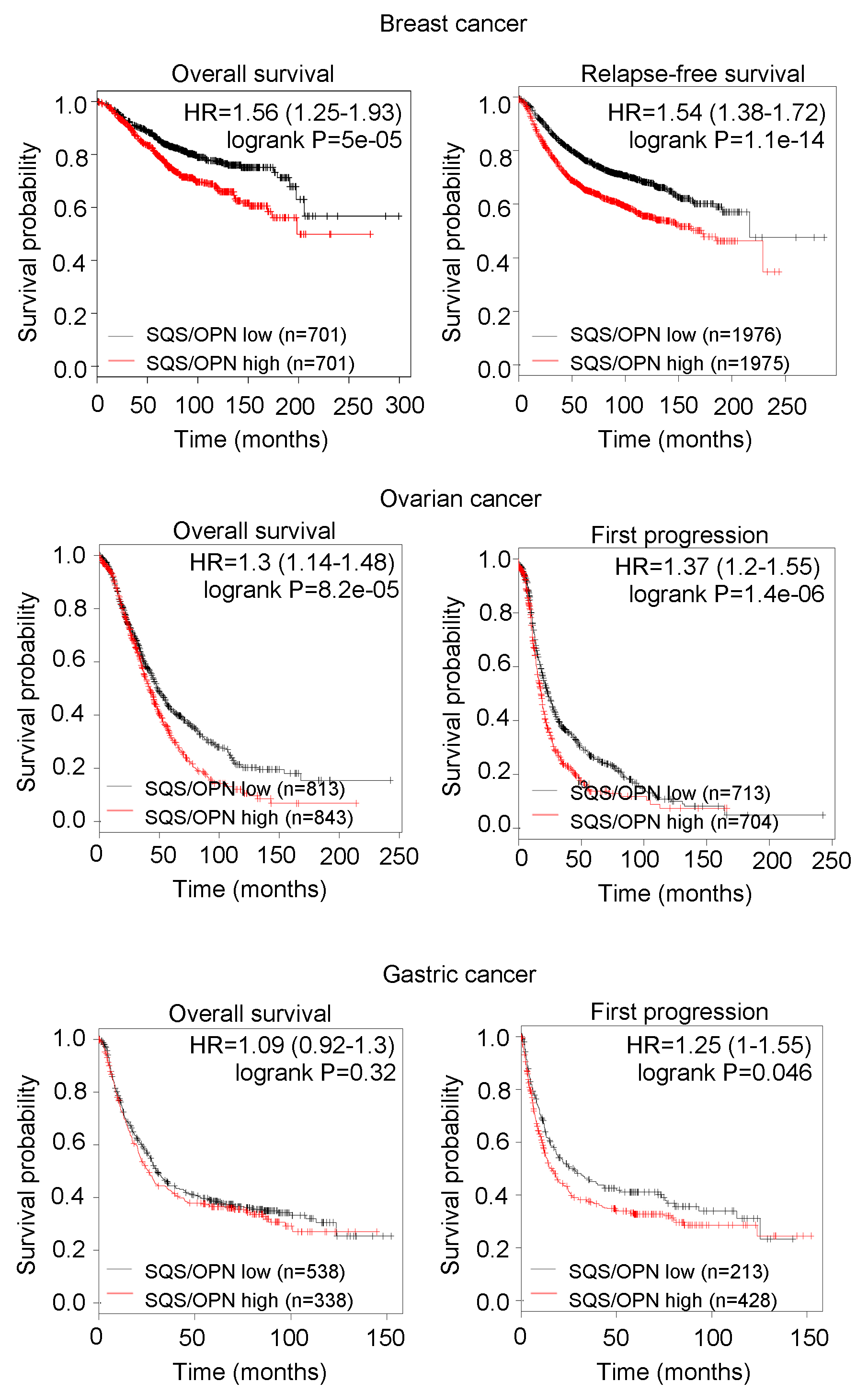


**Figure S6**. **Relative *SQS* and *SPP1* (OPN) expression levels in the multiple cancer clinical cohorts (KM plotter).** (A)Breast cancer. (B) Ovarian cancer. (C) Gastric cancer.

**Table S1. Canonical pathway predicted in FDFT1 (SQS) overexpression transcriptomics**

| Category | Term | Count | % | *P*-Value | Total | Enrichment | Bonferroni | Benjamini | FDR |
| --- | --- | --- | --- | --- | --- | --- | --- | --- | --- |
| KEGG_PATHWAY | hsa04360:Axon guidance | 7 | 1.857 | 0.021 | 127 | 3.213 | 0.983 | 0.983 | 23.056 |
| KEGG_PATHWAY | hsa04151:PI3K-Akt signaling pathway | 12 | 3.183 | 0.032 | 345 | 2.028 | 0.998 | 0.958 | 33.503 |
| KEGG_PATHWAY | hsa04350:TGF-beta signaling pathway | 5 | 1.326 | 0.054 | 84 | 3.470 | 1.000 | 0.972 | 50.141 |
| KEGG_PATHWAY | hsa04530:Tight junction | 5 | 1.326 | 0.060 | 87 | 3.350 | 1.000 | 0.950 | 53.946 |
| KEGG_PATHWAY | hsa04974:Protein digestion and absorption | 5 | 1.326 | 0.062 | 88 | 3.312 | 1.000 | 0.917 | 55.199 |
| KEGG_PATHWAY | hsa04010:MAPK signaling pathway | 9 | 2.387 | 0.066 | 253 | 2.074 | 1.000 | 0.888 | 57.227 |
| KEGG_PATHWAY | hsa05162:Measles | 6 | 1.592 | 0.076 | 133 | 2.630 | 1.000 | 0.886 | 62.529 |
| KEGG_PATHWAY | hsa04550:Signaling pathways regulating pluripotency of stem cells | 6 | 1.592 | 0.090 | 140 | 2.498 | 1.000 | 0.896 | 69.005 |
| KEGG_PATHWAY | hsa04360:Axon guidance | 7 | 1.857 | 0.021 | 127 | 3.213 | 0.983 | 0.983 | 23.056 |

**Table S2. Relationship between OPN expression and clinicopathological factors in 126 lung cancer patients**

|  | OPN expression | | | | |
| --- | --- | --- | --- | --- | --- |
| Characteristics | | Patient  No.  (126) | Low (0,1)  (n =33) | High (2,3)  (n =93) | **P* value |
| Age | |  |  |  | 0.449^†^ |
| Years (mean ± SD) | |  | 59.9 ± 15.4 | 61.7 ± 11.5 |  |
| Sex | |  |  |  | 0.6780^‡^ |
| Male | | 65 | 16 | 49 |  |
| Female | | 61 | 17 | 44 |  |
| Smoking status | |  |  |  | 0.006^‡^* |
| No | | 74 | 26 | 48 |  |
| Yes | | 52 | 7 | 45 |  |
| Histological type | |  |  |  |  |
| Adenocarcinoma | | 77 | 26 | 51 | 0.014^‡^* |
| Squamous cell carcinoma | | 41 | 4 | 37 |  |
| Large cell carcinoma | | 8 | 3 | 5 |  |
| Stage | |  |  |  | 0.005^‡^* |
| I | | 36 | 17 | 19 |  |
| II | | 16 | 3 | 13 |  |
| III | | 35 | 4 | 31 |  |
| IV | | 39 | 9 | 30 |  |
| Tumor status | |  |  |  | 0.186^‡^ |
| T1 | | 26 | 10 | 16 |  |
| T2 | | 61 | 16 | 45 |  |
| T3 | | 7 | 0 | 7 |  |
| T4 | | 32 | 7 | 25 |  |
| Lymph node status | |  |  |  | 0.024^‡^* |
| N0 | | 48 | 18 | 30 |  |
| N1-3 | | 78 | 15 | 63 |  |
| Distal metastasis status | |  |  |  | 0.521^‡^ |
| M0 | | 86 | 24 | 62 |  |
| M1 | | 40 | 9 | 31 |  |
| Recurrence status | |  |  |  | 0.43^‡^ |
| No | | 50 | 15 | 35 |  |
| Yes | | 76 | 18 | 58 |  |
|  | |  |  |  |  |

**P* value < 0.05 was considered statistically significant.

^†^Student’s test for continuous variables.

^‡^Chi-square test for categorical variables.

**Table S3. Cox univariate and multivariate regression analysis of prognostic factors for overall and disease-free survival in 126 lung cancer patients**

| **Cox univariate analysis (OS)** | | |  |  |  |
| --- | --- | --- | --- | --- | --- |
| Variables | | Comparison | HR (95% CI) | | *P*-value |
| T |  | T1-T2; T3-T4 | 2.105 (1.368-3.242) | | 0.001* |
| N |  | N0; N1-N3 | 2.440 (1.547-3.847) | | <0.001* |
| M |  | M0; M1 | 2.742 (1.776-4.234) | | <0.001* |
| OPN |  | High (2,3); Low (0,1) | 1.843 (1.135-2.992) | | 0.013 |
| **Cox multivariate analysis (OS)** | | |  |  |  |
| Variables | | Comparison | HR (95% CI) | | *P*-value |
| T |  | T1-T2; T3-T4 | 1.223 (0.754-1.983) | | 0.415 |
| N |  | N0; N1-N3 | 2.358 (1.468-3.788) | | <0.001* |
| M |  | M0; M1 | 1.713 (1.055-2.780) | | 0.029* |
| OPN |  | High (2,3); Low (0,1) | 1.707 (1.030-2.828) | | 0.038 |
| **Cox univariate analysis (DFS)** | | |  |  |  |
| Variables | | Comparison | HR (95% CI) | | *P*-value |
| T |  | T1-T2; T3-T4 | 2.126 (1.378-3.280) | | 0.001* |
| N |  | N0; N1-N3 | 2.403 (1.559-3.704) | | <0.001* |
| M |  | M0; M1 | 2.524 (1.597-3.898) | | <0.001* |
| OPN |  | High (2,3); Low (0,1) | 1.624 (1.001-2.637) | | 0.05* |
| **Cox multivariate analysis (DFS)** | | |  |  |  |
| Variables | | Comparison | HR (95% CI) | | *P*-value |
| T |  | T1-T2; T3-T4 | 1.279 (0.785-2.086) | | 0.323 |
| N |  | N0; N1-N3 | 1.911 (1.188-3.073) | | 0.008* |
| M |  | M0; M1 | 1.899 (1.165-3.095) | | 0.010* |
| OPN |  | High (2,3); Low (0,1) | 1.365 (0.824-2.262) | | 0.227 |

**Table S4. Correlation between levels of SQS and OPN in 126 lung cancer patients**

|  | SQS^a^ | | | |  |
| --- | --- | --- | --- | --- | --- |
| OPN^b^ | - | 1+ | 2+ | 3+ | Patient  No. |
| - | 4 | 2 | 0 | 2 | 8 |
| 1+ | 12 | 8 | 5 | 0 | 25 |
| 2+ | 14 | 12 | 27 | 13 | 66 |
| 3+ | 4 | 8 | 8 | 7 | 27 |

a: Detection of the expression of SQS and OPN by immunochistochemistry.

b: Intensity of immunohistochemical staining: -, negative; 1+,0-20% of tumor cell stained; 2+,20-50% of tumor cell stained; 3+,>50% of tumor cell stained.

**Supplementary Table 5. KEY RESOURCES TABLE**

| **Antibody** | **Source, catalog number** | **Application** | **Dilution** |
| --- | --- | --- | --- |
| SQS | Genetex,GTX104091 | Western blot, IHC* | 1:2000,1:100* |
| OPN | IBL ,#18625 | Western blot | 1:1000 |
| OPN | Abcam, ab69498 | IHC* | 1:1000 |
| α-tubulin | Sigma-Aldrich, #T5168 | Western blot | 1:5000 |
| MMP1 | Proteintech, #10371-2-AP | Western blot | 1:1000 |
| pSrc | Cell Signaling, #2101 | Western blot | 1:1000 |
| Src | Cell Signaling, #2109 | Western blot | 1:1000 |
| pERK | Cell Signaling, #9101 | Western blot | 1:1000 |
| ERK | Cell Signaling,#9102 | Western blot | 1:1000 |
| pAKT | Cell Signaling,#4060 | Western blot | 1:1000 |
| AKT | Cell Signaling,#4691 | Western blot | 1:1000 |
| CD44 | Cell Signaling,#5640 | Western blot | 1:1000 |
| Abbreviations: IHC: immunohistochemistry | | | |
| **Reagent** | **Source, catalog number** | **Function** | **Concentration** |
| OPN | PEPROTECH, #120-35 | Recombinant protein | 10 μg/mL |
| Src Inhibitor-1 (SKI) | Sigma-Aldrich, S2075 | Src Inhibitor | 10 μM |
| PD-98059 | Sigma-Aldrich, P215 | MAPK Inhibitor | 20 μM |
| LY294002 | Sigma-Aldrich, L9908 | PI3K Inhibitor | 10 μM |
| Methyl-β-cyclodextrin | Sigma-Aldrich, C4555 | depletion of cholesterol | 10mM |
| Cholesterol | Sigma-Aldrich, C3045 | - | 10,100 μg/mL |
| **shRNA Target Sequence** | | | |
| **Gene symbol** | **Target Sequence** | **Region** | **Score** |
| SQS_clone1 | CCTACTACTTTAATCCCTA | ORF | ORIGENE |
| SQS_clone2 | CTGTCAAAGCCATCATATA | ORF | ORIGENE |
| OPN_clone1 | CATCTTCTGAGGTCAATTA | ORF | ORIGENE |
| Abbreviations: ORF: open reading frame; CDS: Coding Sequence; three prime untranslated region | | | |
| **Primer Sequence** | | | |
| **Gene** | **Sequence** | | |
| SPP1(OPN)-forward | ATC TCC TAG CCC CAC AGA CCC TT | | |
| SPP1(OPN)-reverse | TCG GTT GCT GGC AGG TCC GT | | |
| S26-forward | CCG TGC CTC CAA GAT GAC AAA G | | |
| S26-reverse | ACT CAG CTC CTT ACA TGG GCT T | | |
| FDFT1(SQS)-forward | ACC CCT TAG TTG GTG AAG ATA CAG | | |
| FDFT1(SQS)-reverse | GTG CAG TGC ATT GGT TAT AAG TTC | | |
| MMP1-forward | TGA GGG GAA CCC TCG CTG GG | | |
| MMP1-reverse | TGT CCC GAT GAT CTC CCC TGA CA | | |
| GAPDH-forward | GTC CAC TGG CGT CTT CAC CAC C | | |
| GAPDH-reverse | AGG CAT TGC TGA TGA TCT TGA GGC | | |
